# A Telescoped Continuous Flow Enantioselective Process for Accessing Intermediates of 1-Aryl-1,3-diols as Chiral Building Blocks

**DOI:** 10.1021/acs.joc.3c02040

**Published:** 2023-10-16

**Authors:** Aitor Maestro, Bence S. Nagy, Sándor B. Ötvös, C. Oliver Kappe

**Affiliations:** † Department of Organic Chemistry I, University of the Basque Country, UPV/EHU, Paseo de la Universidad 7, 01006 Vitoria-Gasteiz, Spain; ‡ Institute of Chemistry, University of Graz, NAWI Graz, A-8010 Graz, Austria; § Center for Continuous Flow Synthesis and Processing (CC FLOW), Research Center Pharmaceutical Engineering GmbH (RCPE), A-8010 Graz, Austria

## Abstract

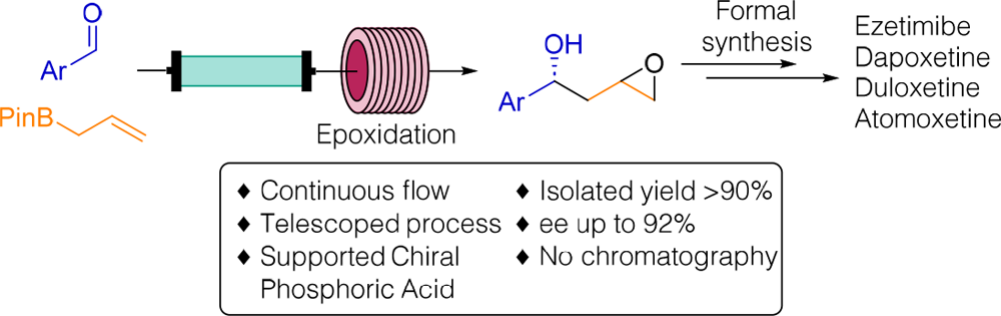

A telescoped continuous flow process is reported for the enantioselective synthesis of chiral precursors of 1-aryl-1,3-diols, intermediates in the synthesis of ezetimibe, dapoxetine, duloxetine, and atomoxetine. The two-step sequence consists of an asymmetric allylboration of readily available aldehydes using a polymer-supported chiral phosphoric acid catalyst to introduce asymmetry, followed by selective epoxidation of the resulting alkene. The process is highly stable for at least 7 h and represents a transition-metal free enantioselective approach to valuable 1-aryl-1,3-diols.

1-Aryl-1,3-diols **1** are important synthetic building blocks for the pharmaceutical industry.^1^ They are key intermediates in the synthesis of numerous drugs, including ezetimibe (treatment of high blood cholesterol),^2^ dapoxetine (premature ejaculation),^3^ atomoxetine (attention deficit hyperactivity disorder),^4^ and duloxetine (major depressive and anxiety disorders)^5^ (Figure 1).

**Figure 1:**
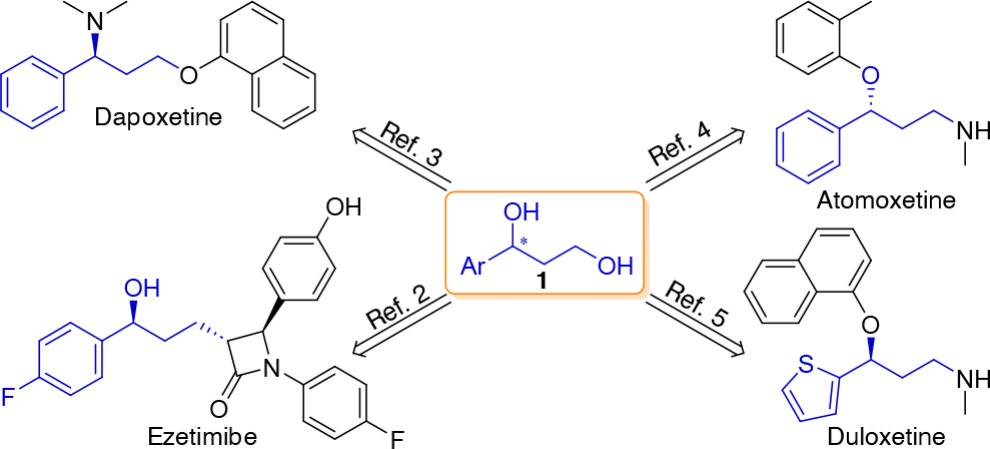
Relevant drugs synthesized from 1-aryl-1,3-diols **1**.

Several synthetic routes have been developed to access optically active 1-aryl-1,3-diols using enantioselective reactions^5,6^ and, most interestingly, organocatalysis.^7^ While asymmetric catalytic methods are more atom-efficient and produce less waste, the high cost of chiral ligands and organocatalysts often makes chiral auxiliaries the preferred option.^2,3,8^ To maximize the efficiency of existing catalytic enantioselective transformations, there has been a growing interest in the development of recyclable catalysts during the past decade.^9^ In particular, chiral phosphoric acids (CPAs) have seen widespread adoption due to their versatility.^10^ Numerous applications of immobilized chiral CPAs have been reported to date, highlighting their significant potential to facilitate catalyst recovery.^11^

With regard to CPA-catalyzed enantioselective reactions with potential to synthesize optically active precursors of 1,3-diols **1**, Antilla and co-workers reported a highly enantioselective approach for allylboration of aldehydes using a 2,4,6-tris-isopropyl-derived CPA,^12^ known as TRIP^13^ (Scheme 1A). A few years later, a copolymerization-based strategy was employed to immobilize TRIP onto a polystyrene resin, and the resulting supported catalyst (PS-TRIP) was successfully applied to enantioselective allylboration reactions as a highly recyclable organocatalyst.^11^ Even though some of the immobilized CPAs have been shown to be exceptionally active and robust,^11b,11d^ they have not been widely utilized for the enantioselective synthesis of active pharmaceutical ingredients (APIs) and related compounds.^14^

**Scheme 1:**
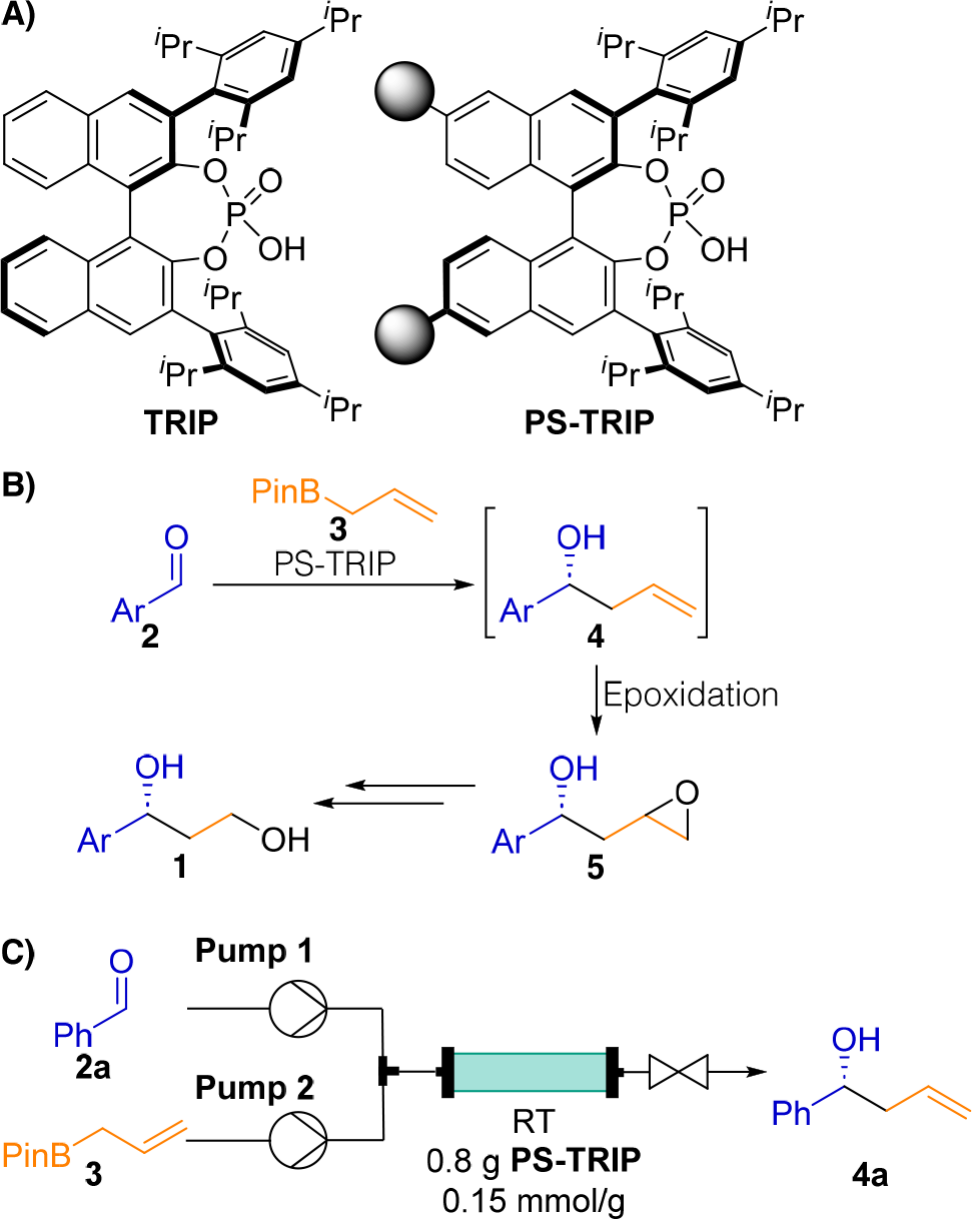
(A) CPAs Used in the Enantioselective Allylboration of Aldehydes, (B) Proposed Synthetic Route to 1-Aryl-1,3-diols, and (C) Continuous Flow Setup Used for the Allylboration Step

Due to improved productivity, easier scalability, and waste reduction compared to more conventional batch procedures, telescoped continuous flow processes involving immobilized chiral catalysts have proven to be particularly useful for the multistep synthesis of optically active targets.^15^ Building on our previous efforts in flow synthesis of chiral APIs and their advanced intermediates,^16^ we hypothesized that merging PS-TRIP-catalyzed asymmetric allylboration with selective epoxidation of the resulting chiral alkene in an uninterrupted flow process would open a simple and efficient entry to optically active 1,3-diols as key intermediates of atomoxetine, dapoxetine, duloxetine, and ezetimibe. The planned two-step process would produce enantioenriched epoxy alcohols **5** from readily available nonchiral aldehydes, which can then be easily transformed into the desired chiral diols **1** (Scheme 1B).^17^ By carefully selecting reaction conditions, we aimed to eliminate the need for any chromatographic purification, thereby facilitating larger-scale syntheses.

Our study began with optimizing the parameters of individual reaction steps. The activity of the PS-TRIP catalyst for asymmetric allylboration was explored in a flow setup consisting of two separate reagent feeds: solutions of benzaldehyde **2a** (1.0 equiv) and allylboronic ester **3** (1.2 equiv). The reagent streams were pumped at a flow rate of 100 μL/min each and were combined before entering a packed bed reactor containing 0.8 g of the supported catalyst (Scheme 1C). This corresponded to a residence time on the catalyst bed of ∼15 min. Several solvents were evaluated with the purpose of making the overall process greener.^18^ The effect of substrate concentration was also explored to maximize the productivity. The best results for obtaining alkene **4a** were achieved in 97% yield and 90% enantiomeric excess (ee) using a substrate concentration of 0.15 M in toluene as the solvent (see Table S1 for details).

Next, various strategies were explored for the subsequent epoxidation, initially under batch conditions (Table 1). We found that hydrogen peroxide as an oxidant resulted in overoxidation of the desired chiral alcohol (**5a**) to the corresponding ketone **6a**, making the process unsuitable for further development (Table 1, entry 1). Dimethyldioxirane (DMDO), generated from acetone and Oxone (2KHSO_5_·KHSO_4_·K_2_SO_4_) in a buffered aqueous solution,^19^ showed high conversion and selectivity (Table 1, entry 2) but involved miscibility issues with toluene. To avoid solubility problems that could affect the reactivity in flow, we next evaluated organic peracids. Commercially available solutions of peracetic acid (PAA) showed high selectivity but only poor conversion (Table 1, entries 3 and 4).

**Table 1:**
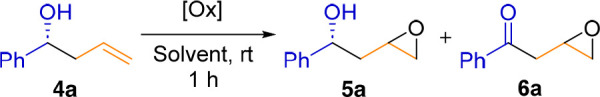
Optimization of the Epoxidation of **4a** under Batch Conditions^a^

entry	oxidant (equiv)	solvent	**5a** (%)	**6a** (%)
1	H_2_O_2_ (1.2)	2:1 acetone/H_2_O	54	26
2	DMDO (2.0)	2:1 acetone/H_2_O	96	1
3	PAA (4.0)	toluene	25	nd
4	PAA (8.0)	toluene	30	nd
5	*m*CPBA (2.0)	toluene	62	nd
6	*m*CPBA (3.0)	toluene	80	nd
7	*m*CPBA (4.0)	toluene	93	nd

a General conditions: **4a** (0.1 mmol, 1 equiv), oxidant, solvent (1.0 mL). The yields were determined by HPLC area %. nd, not detected.

Although the *in situ* generation of peracids under continuous flow conditions is well-known,^20^ preliminary tests showed significant overoxidation to ketone **6a**, probably due to the large excess of H_2_O_2_ required in these reactions. Therefore, we finally tested *m*-chloroperbenzoic acid (*m*CPBA) as the epoxidation agent. Gratifyingly, excellent conversion and selective epoxidation were achieved in the presence of 4.0 equiv of *m*CPBA, making it the preferred oxidant for further development (Table 1, entries 5–7). Although the diastereoselectivity of the epoxidation process is minimal, the late removal of the chiral center on the epoxide makes it not relevant for synthesis of diols **1**. The *m*CPBA-mediated selective epoxidation was then transferred to continuous flow using a simple coil reactor, ensuring conversions of ≥90% within residence times of ∼10 min at 85 °C (see Table S2 for details).

Following step-by-step optimization, we combined the PS-TRIP-catalyzed asymmetric allylboration of benzaldehyde (**2a**) and the subsequent epoxidation in a telescoped flow sequence to access epoxy alcohol **5a**, a chiral intermediate of atomoxetine and dapoxetine (Scheme 2A). Downstream to the packed bed reactor, the *m*CPBA feed served a double role. Apart from functioning as an epoxidation agent, it also quenched any unreacted allyl pinacol ester, thereby preventing racemic background reactions in the case of uncompleted allylboration. To safely quench any excess oxidant, the outlet of the reactor was directed into a stirred solution of Na_2_S_2_O_5_. With the optimized setup in hand, we performed a continuous long run for 7 h. The overall process was followed by off-line HPLC with samples taken and analyzed every hour. We were pleased to find no decrease in either the conversion or enantioselectivity, showing the robustness of the process (Scheme 2B). Contrary to previous reports on enantioselective allylboration reactions,^11d,12,21^ the process presented here did not require any chromatographic purification but a simple acid/base extractive workup to isolate the desired chiral adduct in sufficiently pure form.

**Scheme 2:**
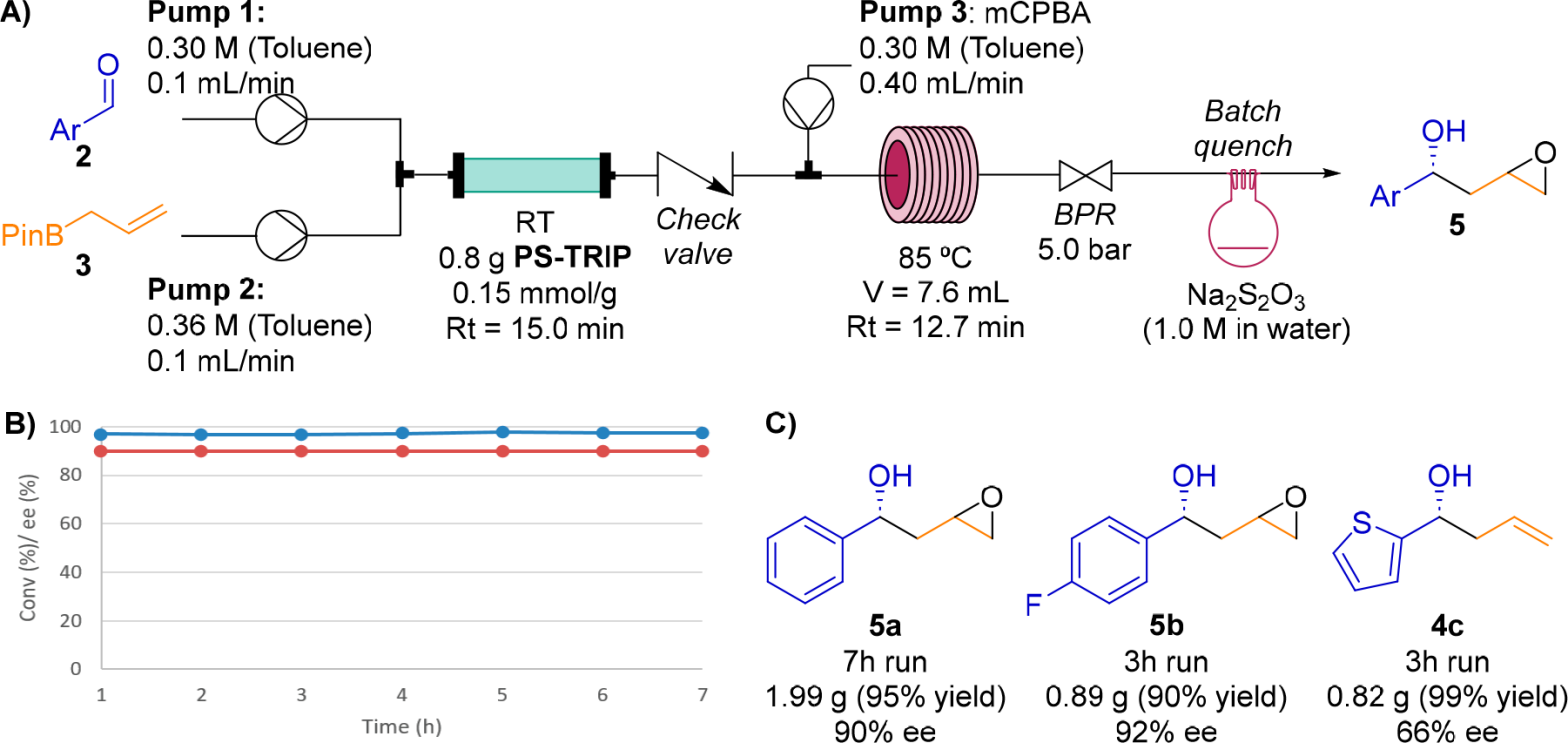
(A) Optimal Setup for the Telescoped Asymmetric Allylboration/Epoxidation Process, (B) Yields (blue) and ee (red) of **5a** over Time (HPLC), and (C) Chiral Intermediates for the Synthesis of APIs

To obtain potential precursors of ezetimibe and duloxetine, the two-step flow synthesis was next attempted using 4-fluorobenzaldehyde (**2b**) and 2-thiophenecarboxaldehyde (**2c**) as the substrate, respectively (Scheme 2C). Epoxy alcohol **5b** was smoothly produced from aldehyde **2b** during a continuous 3 h run (90% yield, 92% ee) under conditions identical to those applied in the synthesis of **3a**. In the targeted synthesis of oxirane **5c** from aldehyde **2c**, the epoxidation step resulted in a complex mixture, probably due to the polymerization of the thiophene ring.^22^ In this case, the process was stopped after the allylboration step (performed using the setup shown in Scheme 1C; see also the Supporting Information for details) to afford alkene **4c** in 99% yield and 66% ee.

To illustrate the applicability of epoxides **5** in the synthesis of 1-aryl-1,3-diols **1**, we performed the ring opening of epoxide **5a** in acidic media, affording triol **7a** in high yield (Scheme 3). Further transformations of triols **7** to the corresponding diols **1** are known in the literature.^17^

**Scheme 3:**
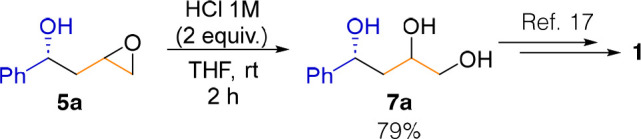
Formal Synthesis of 1-Aryl-1,3-diols **1**

In summary, we have developed a telescoped continuous flow process using an immobilized CPA-mediated enantioselective allylboration as the key step followed by *m*CPBA-mediated selective alkene epoxidation. Our strategy consists of a transition-metal free catalytic method to access triols **7** and diols **1** in high yield and enantiocontrol by using a robust immobilized organocatalyst. By exploiting an uninterrupted flow process, we obtained chiral epoxides **5** in a simple and efficient manner, without the need for any chromatographic purification. With a cumulative residence time of <30 min, the protocol enabled a notable chemical intensification compared to earlier methodologies.

## Experimental Section

### General Information

All solvents and chemicals were obtained from typical commercial vendors and used as received, without any further purification. ^1^H, ^19^F, and ^13^C NMR spectra were recorded on a Bruker Avance III 300 MHz instrument at room temperature, in CDCl_3_ as the solvent, at 300 and 75 MHz. Chemical shifts (δ) are reported in parts per million relative to the residual solvent peak (CDCl_3_, ^1^H, 7.26 ppm; ^13^C, 77.16 ppm). Coupling constants are reported in hertz. Multiplicity is reported with the usual abbreviations.

When required, column chromatographic purification was performed by using a Biotage Isolera automated flash chromatography system with cartridges packed with KP-SIL, 60 Å (32–63 μm particle size). Analytical thin-layer chromatography (TLC) was carried out using Merck silica gel 60 GF254 plates. Compounds were visualized by means of ultraviolet (UV) or KMnO_4_.

Analytical HPLC analysis was carried out on a C18 reversed-phase (RP) analytical column (150 mm × 4.6 mm, particle size of 5 mm) at 37 °C by using mobile phases A [90:10 (v/v) water/acetonitrile with 0.1% TFA] and B (acetonitrile with 0.1% TFA) at a flow rate of 1.5 mL/min. The following gradient was applied: linear increase from 3% to 5% B over 3 min, linear increase from 5% to 30% B over 4 min, linear increase from 30% to 100% B over 3 min, hold at 100% B for 2 min, linear decrease from 100% to 3% B over 0.5 min, and hold at 3% B for 2.5 min.

The ee of the compounds was determined by chiral HPLC or chiral GC. Chiral HPLC analysis was performed on a Shimadzu HPLC system (DGU-403 degassing unit, CTO-40S column oven, CBM20 system controller, SPD-40 UV–visible detector, LC-20AT pumps). Chiral GC analysis was performed on a Trace-GC (ThermoFisher) GC system equipped with a flame ionization detector (FID), using an Rt-BDEXse column [30 m × 0.32 mm (inside diameter) × 0.25 μm df] (Restek GmbH) and helium as a carrier gas (linear velocity of 0.5 mL min^–1^). FID was used for detection, and the detector gases used for flame ionization were hydrogen and synthetic air (5.0 quality).

Optical rotation was measured in CHCl_3_ (HPLC-grade) at 25 °C against the sodium D line (λ = 589 nm) on a PerkinElmer Polarimeter 341 using a 10 cm path length cell. The specific rotation was calculated with the following equation
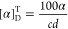
where *T* is the temperature in degrees Celsius, *D* is the sodium D line emission, α is the angle of rotation, *c* is the concentration of the solution in grams per 100 mL, and *d* is the length of the polarimeter tube in decimeters (here 1 dm). The given data were calculated as the average of three measurements. The absolute configuration was determined by comparison of the optical rotation for compound **4c**, and the absolute configurations of other compounds were assigned by analogy.^12^

High-resolution mass spectra were recorded in either negative or positive mode on an Agilent 6230 TOF LC/MS instrument (G6230B) by flow injections on an Agilent 1260 Infinity Series HPLC instrument (HiP degasser G4225A, binary pump G1312B, ALS autosampler G1329B, TCC column thermostat G1316A, and DAD detector G4212B).

Equipment for the continuous flow reactions was assembled using commercially available components. Liquid streams were pumped by using Syrris Asia syringe pumps. Flow systems were pressurized by using an adjustable backpressure regulator (BPR) from Zaiput and/or by using a fixed-pressure BPR from IDEX. Reaction coils were heated by means of a conventional oil bath. Reagent feeds were streamed directly or by using injection valves and sample loops. Sample loops and reactor coils were made by using perfluoroalkoxy alkane (PFA) tubings (1/16 in. outside diameter, 0.80 mm inside diameter or 1/8 in. outside diameter, 1.58 mm inside diameter). Details of reaction setups as well as general procedures can be found in the following sections.

### Synthesis of the Catalysts

The synthesis of PS-TRIP catalysts was performed following Pericàs’s procedure.^11d,11g^ The catalyst loading of the resin was calculated on the basis of the P elemental analysis by using the following formula:
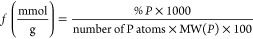
Anal. P, 0.48%; *f* = 0.15 mmol/g.

### General Procedure for the Batch Synthesis of Racemic **4**

The corresponding aldehyde (1.0 mmol, 1.0 equiv) was dissolved in 5 mL of toluene, and AllylBpin (225.1 μL, 1.2 mmol, 1.2 equiv) was added dropwise at room temperature. The reaction mixture was stirred overnight and then concentrated under vacuum. The reaction crude was purified by column chromatography on silica gel (1:0 to 7:3 hexanes/Et_2_O, followed at 210 nm). The reported data match the literature.

### General Procedure for the Batch Synthesis of Racemic **5**

The corresponding aldehyde (1.0 mmol, 1.0 equiv) was dissolved in 5 mL of toluene, and AllylBpin (225.1 μL, 1.2 mmol, 1.2 equiv) was added dropwise at room temperature. The reaction mixture was stirred overnight, *m*CPBA (75% purity, 739.6 mg, 3.0 mmol, 3.0 equiv) added in one portion, and the mixture stirred overnight at room temperature. The crude reaction was quenched with 1.0 M Na_2_S_2_O_3_ (10 mL), and the organic phase was washed with 1.0 M NaOH (3 × 10 mL), saturated NaHCO_3_ (1 × 10 mL), and brine (1 × 10 mL). The organic phase was dried over MgSO_4_, filtered, and concentrated.

### General Procedure for the Batch Synthesis of **7**

Oxirane **5a** (82.1 mg, 0.5 mmol, 1.0 equiv) was dissolved in 1 mL of THF, and 1.0 M HCl (0.75 mL, 0.75 mmol, 1.5 equiv) was added dropwise. The reaction mixture was stirred for 2 h at room temperature, diluted with Et_2_O (10 mL), extracted, and washed with brine (1 × 10 mL). The crude reaction mixture was purified by column chromatography on silica gel (1:0 to 1:1 hexanes/Et_2_O, followed at 210 nm).

### Experimental Procedure for the Telescoped Flow Synthesis of Oxiranes **5**

First, 0.8 g of the PS-TRIP catalyst was loaded into an adjustable Omnifit glass column [10 mm (inside diameter)]. Prior to the reactions, the catalyst bed was swollen by pumping toluene at a rate of 200 μL/min for 30 min. Stock solutions (in toluene) of aldehyde **2a** (0.30 M, 100 μL/min, 1.0 equiv) and **3** (0.36 M, 100 μL/min, 1.2 equiv) were pumped independently (overall flow rate of 200 μL/min) and combined at room temperature just before the catalyst-containing Omnifit column by using a Syrris Asia syringe pump. A check-valve was added to the exit of the packed bed reactor to avoid back flow. Then, a *m*CPBA^a^ solution in toluene (0.30 M, 400 μL/min, 4.0 equiv) was combined in the reaction coil heated to 85 °C in an oil bath.^b^ The system was pressurized at 5 bar by using a Zaiput BPR. The reaction outcome was quenched by collecting the mixture directly into a stirred aqueous solution of 1.0 M Na_2_S_2_O_3_. More detailed information about the flow setup is shown in section S3 of the Supporting Information.

*Workup for 1 h of the Reaction in Continuous Flow*. The reaction mixture was collected over 30 mL of a 1.0 M solution of Na_2_S_2_O_3_. The organic phase was then separated and washed with 1.0 M NaOH (3 × 50 mL), saturated NaHCO_3_ (1 × 50 mL), and brine (1 × 50 mL). The organic phase was then dried over MgSO_4_, filtered, and concentrated under vacuum.

*Workup for 3 h of the Reaction in Continuous Flow*. The reaction mixture was collected over 90 mL of a 1.0 M solution of Na_2_S_2_O_3_. The organic phase was then separated and washed with 1.0 M NaOH (3 × 150 mL), saturated NaHCO_3_ (1 × 150 mL), and brine (1 × 150 mL). The organic phase was then dried over MgSO_4_, filtered, and concentrated under vacuum.

Note that the epoxidation step was not suitable for the synthesis of compound **5c** due to the undesired polymerization of the thiophene in the reaction.^22^ In contrast to compounds **5a** and **5b**, in which colorless or pale yellow solutions were observed, during the synthesis of **5c**, a black precipitate is formed in the case of **4c** and *m*CPBA, leading to the clogging of the system and a complex mixture of byproducts.

Therefore, the 3 h run for the synthesis of **4c** was performed by collecting the allylation product of **2a** and **3** just after the packed bed reactor, using the optimal reaction conditions for the allylboration reaction (Table 1, entry 6). The reaction outcome was quenched by collecting directly into a stirred 1.0 M Na_2_S_2_O_3_ (90 mL), and the organic phase was extracted in toluene, dried over MgSO_4_, filtered, and concentrated. The reaction crude was purified by column chromatography on silica gel (1:0 to 7:3 hexanes/Et_2_O, followed at 210 nm).

### Characterization Data of **4**, **5**, and **7**

#### (*R*)-1-(Thiophen-2-yl)but-3-en-1-ol (**4c**)

The product was synthesized using the flow procedure and collected for 3 h after the steady state, affording 0.82 g (99%) of the product as a colorless oil. The reported data match the literature:^12^^1^H NMR (300 MHz, CDCl_3_) δ 7.27 (dd, *J* = 4.2, 2.2 Hz, 1H), 7.05–6.96 (m, 2H), 5.85 (ddt, *J* = 17.2, 10.2, 7.1 Hz, 1H), 5.25–5.14 (m, 2H), 5.00 (td, *J* = 6.5, 2.7 Hz, 1H), 2.70–2.56 (m, 2H), 2.37 (bs, 1H); ^13^C{^1^H} NMR (75 MHz, CDCl_3_) δ 147.9, 134.0, 126.7, 124.7, 123.8, 118.9, 69.5, 43.9; HRMS (TOF+) *m*/*z* [2M + K]^+^ calcd for C_16_H_20_O_2_S_2_K 347.0537, found 347.0571;  (*c* = 1.07 in CHCl_3_). Literature data for (*S*)-**4c** (96% ee): −12.33 (*c* = 1.07 in CHCl_3_).^12^ For chiral GC-FID analysis, the oven was heated to 60 °C before injection of the sample and held at that temperature for 1.0 min after the injection. Then, the temperature was increased to 130 °C at a rate of 5.0 °C/min and held for 10.0 min. The temperature was then increased to 145 °C at a rate of 3.0 °C/min and held for 1.0 min. *t*_R_ = 28.4 (minor) and 28.5 min (major).

#### (1*R*)-2-(Oxiran-2-yl)-1-phenylethan-1-ol (**5a**)

The product was synthesized using the telescoped flow procedure and collected for 7 h after the steady state, affording 1.99 g (95%) of the product as a yellowish oil (mixture of diastereomers): ^1^H NMR (300 MHz, CDCl_3_) δ 7.52–7.28 (m, 10H), 5.11–4.83 (m, 2H), 3.18 (dtd, *J* = 6.8, 4.0, 2.7 Hz, 1H), 3.03 (dtd, *J* = 7.0, 4.1, 2.8 Hz, 1H), 2.84 (dd, *J* = 4.8, 4.0 Hz, 1H), 2.76 (dd, *J* = 4.8, 4.0 Hz, 1H), 2.62 (dd, *J* = 4.8, 2.8 Hz, 1H), 2.51 (dd, *J* = 5.0, 2.8 Hz, 1H), 2.42 (bs, 1H). 2.22–2.01 (m, 2H), 1.97–1.75 (m, 2H); ^13^C{^1^H} NMR (75 MHz, CDCl_3_) δ 144.2, 143.9, 128.7, 128.7, 127.9, 127.8, 125.9, 125.7, 72.9, 71.9, 50.4, 50.1, 47.2, 46.9, 41.9, 41.4; HRMS (TOF+) *m*/*z* [M + H]^+^ calcd for C_10_H_12_O_2_H 165.0910, found 165.0902;  (*c* = 1.05 in CHCl_3_); HPLC (AD-H, 5/95 i-PrOH/*n*-heptane, flow rate of 1.0 mL/min, oven temperature of 12 °C, λ = 210 nm): *t*_R_ = 18.5 (diastereomer 1, major), 20.5 (diastereomer 2, major), 21.7 (diastereomer 1, minor), and 23.3 min (diastereomer 2, minor).

#### (1*R*)-1-(4-Fluorophenyl)-2-(oxiran-2-yl)ethan-1-ol (**5b**)

The product was synthesized using the telescoped flow procedure and collected for 3 h after the steady state, affording 0.89 g (90%) of the product as a yellowish oil (mixture of diastereomers): ^1^H NMR (300 MHz, CDCl_3_) δ 7.37–7.27 (m, 4H), 7.09–6.95 (m, 4H), 4.94–4.81 (m, 2H), 3.12 (dtd, *J* = 6.8, 4.0, 2.8 Hz, 1H), 2.94 (dtd, *J* = 7.0, 4.1, 2.7 Hz, 1H), 2.88 (s, 2H), 2.78 (dd, *J* = 4.8, 4.1 Hz, 1H), 2.72 (dd, *J* = 4.9, 4.0 Hz, 1H), 2.56 (dd, *J* = 4.8, 2.8 Hz, 1H), 2.46 (dd, *J* = 4.9, 2.7 Hz, 1H), 2.15–1.93 (m, 2H), 1.87–1.65 (m, 2H); ^13^C{^1^H} NMR (75 MHz, CDCl_3_) δ 162.3 (d, *J* = 245.6 Hz), 162.2 (d, *J* = 245.4 Hz), 139.9 (d, *J* = 3.1 Hz), 139.7 (d, *J* = 3.2 Hz), 127.5 (d, *J* = 8.2 Hz), 127.3 (d, *J* = 8.1 Hz), 115.4 (d, *J* = 21.4 Hz), 115.4(2) (d, *J* = 21.4 Hz), 72.1, 71.1, 50.3, 50.0, 47.1, 46.9, 41.9, 41.4; ^19^F NMR (282 MHz, CDCl_3_) δ −114.7, −115.0; HRMS (TOF+) *m*/*z* [M]^+^ calcd for C_10_H_11_FO_2_H 182.0743, found 182.0095;  (*c* = 1.02 in CHCl_3_); HPLC (AD-H, 2/98 i-PrOH/*n*-heptane, flow rate of 1.0 mL/min, oven temperature of 12 °C, λ = 210 nm): *t*_R_ = 37.7 (diastereomer 1, major), 40.9 (diastereomer 2, major), 43.0 (diastereomer 1, minor), and 45.1 min (diastereomer 2, minor).

#### (4*R*)-4-Phenylbutane-1,2,4-triol (**7a**)

The general procedure was followed, affording 72.0 mg (79%) of the product as a yellowish oil (mixture of diastereomers). The reported data match the literature:^23^^1^H NMR (300 MHz, CDCl_3_) δ 7.50–7.20 (m, 10H), 5.06 (t, *J* = 6.0 Hz, 1H), 4.97 (dd, *J* = 8.8, 4.4 Hz, 1H), 4.18–3.99 (m, 2H), 3.69–3.47 (m, 4H), 3.45–2.53 (m, 4H), 2.11–1.84 (m, 4H); ^13^C{^1^H} NMR (75 MHz, CDCl_3_) δ 144.1, 143.9, 128.8, 128.7, 128.1, 127.8, 125.8, 125.6, 74.5, 72.0, 71.5, 69.0, 50.0, 49.7, 42.7, 42.1; HRMS (TOF-) *m*/*z* [M – H]^−^ calcd for C_10_H_13_O_3_ 181.0870, found 181.0879;  (*c* = 0.99 in CHCl_3_); HPLC (AD-H, 5/95 i-PrOH/*n*-heptane, flow rate of 1.0 mL/min, oven temperature of 12 °C, λ = 210 nm): *t*_R_ = 24.7 (diastereomer 1, major), 26.1 (diastereomer 21, minor), 33.2 (diastereomer 2, minor), and 38.5 min (diastereomer 2, major).

## Data Availability

The data underlying this study are available in the published article and its Supporting Information.

## Supporting Information Available

General procedures and characterization data (PDF)

## Footnotes

a During the long run reaction, a color change in the *m*CPBA stock solution was observed, going from a colorless solution to a slightly yellow solution, indicating partial degradation. To avoid a potential conversion decrease during the epoxidation step, a new *m*CPBA solution was prepared after 5 h.

b Higher temperatures resulted in degradation of the *m*CPBA and led to undesired byproducts.
