# Supplementary material for: A Telescoped Continuous Flow Enantioselective Process for Accessing Intermediates of 1-Aryl-1,3-diols as Chiral Building Blocks
Source: J Org Chem. 2023 Oct 16;88(21):15523–9. doi: 10.1021/acs.joc.3c02040 (PMC10629223; doi:10.1021/acs.joc.3c02040)

Supporting information

A Telescoped Continuous Flow Enantioselective Process for Accessing Intermediates of 1-Aryl-1,3-diols as Chiral Building Blocks

Aitor Maestro*[a,b] Bence S. Nagy,[b] Sándor B. Ötvös,[b,c] C. Oliver Kappe*[b,c]

[a] Department of Organic Chemistry I, University of the Basque Country, UPV/EHU, Paseo de la Universidad 7, 01006 Vitoria-Gasteiz, Spain; [b] Institute of Chemistry, University of Graz, NAWI Graz, A-8010 Graz, Austria; [c] Center for Continuous Flow Synthesis and Processing (CC FLOW), Research Center Pharmaceutical Engineering GmbH (RCPE), A-8010 Graz, Austria

Email: aitor.maestro@ehu.eus, oliver.kappe@uni-graz.at

Optimization of the asymmetric allylboration step in flow SI-2

Optimization of the epoxidation in flow SI-3

Experimental set up for the telescoped flow synthesis of oxiranes **5** SI-4

NMR and HPLC data of **4**, **5** and **7** SI-5

NMR spectra of **4**, **5** and **7** SI-12

Optimization of the asymmetric allylboration step in flow

0.8 g of PS-TRIP catalyst was loaded into an adjustable Omnifit® glass column (10 mm ID). Prior to the reactions, the catalyst bed was swollen by pumping the corresponding solvent (see Table below) at 200 µL/min for 30 min. The system was pressurized at 5 bar by using a Zaiput BPR. The stock solutions of aldehyde **2a** (1.0 equiv.) and **3** (1.2 equiv.) were pumped independently (100 µL/min each, 200 µL/min overall flow rate) and combined just before the catalyst-containing Omnifit column by using a Syrris® Asia syringe pump. In each run, the product stream was collected for 10 min after reaching steady state. Between each experiment, the catalyst bed was washed with toluene (200 µL/min for 30 min). The resulting material was diluted in iPrOH analyzed by (chiral) HPLC.

Table S1: General set up for the allylboration reaction in flow.

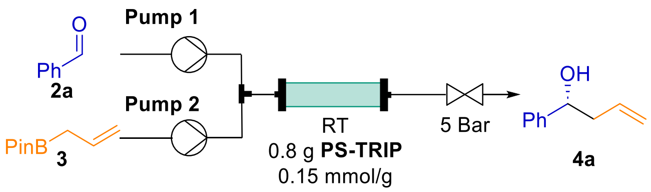

Entry	[M]	Solvent	Yield (%)[a]	ee (%)[b]
1	0.1	Toluene	90	90
2	0.1	Anisole	96	80
3	0.1	2-MeTHF	Trace	-
4	0.1	EtOAc	40	50
5	0.1	Acetone	56	26
6	0.15	Toluene	97	90
7	0.2	Toluene	96	85
8	0.3	Toluene	95	82

a) Yield was determined by HPLC area %.
b) ee was determined by chiral HPLC.

Optimization of the epoxidation in flow

Prior to the reactions, the reaction coil was filled with toluene. The system was pressurized at 5 bar by using a Zaiput BPR. The stock solutions of **4a** (0.15 M in Toluene, 1.0 equiv.) and mCPBA were pumped independently and combined just before the heated reaction coil by using a Syrris® Asia syringe pump. In each run, the product stream was collected for 10 min after reaching steady state. Between each experiment, the reactor was washed with toluene. The resulting material was diluted in MeCN analyzed by HPLC.

Table S2: General set up for the epoxidation in flow.

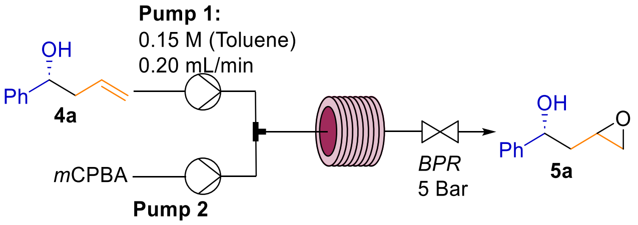

Entry	[M] AllylB	[M] mCPBA	Equiv.	T (ºC)	Flow rate (mL/min)	V (mL)	RT (min)	Yield (%)[a]
1	0.03	0.15	5.0	23	0.8	20	25	25
2	0.03	0.15	5.0	100	1.0	20	20	Messy
3	0.03	0.15	5.0	80	1.0	20	20	99
4	0.03	0.075	2.5	80	1.0	20	20	75
5	0.03	0.09	3.5	80	1.0	20	20	88
6	0.03	0.12	4.0	80	1.0	20	20	91
7	0.015	0.06	4.0	80	2.0	20	10	60
8	0.03	0.12	4.0	80	1.0	11	11	80
9	0.05	0.20	4.0	85	0.6	4.5	7.5	90
10	0.05	0.20	4.0	85	0.6	7.6	12.7	97

a) Yield was determined by HPLC area %.

Experimental set up for the telescoped flow synthesis of oxiranes 5

S3: Optimized set up for the telescoped allylboration-epoxidation process.

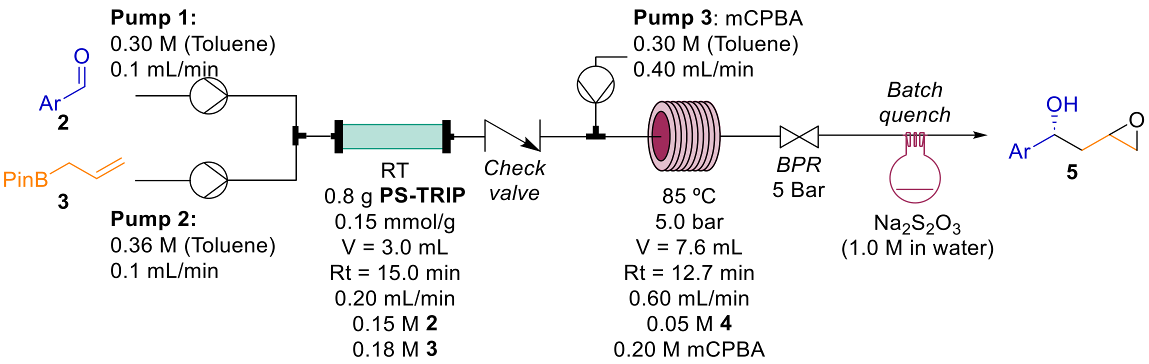

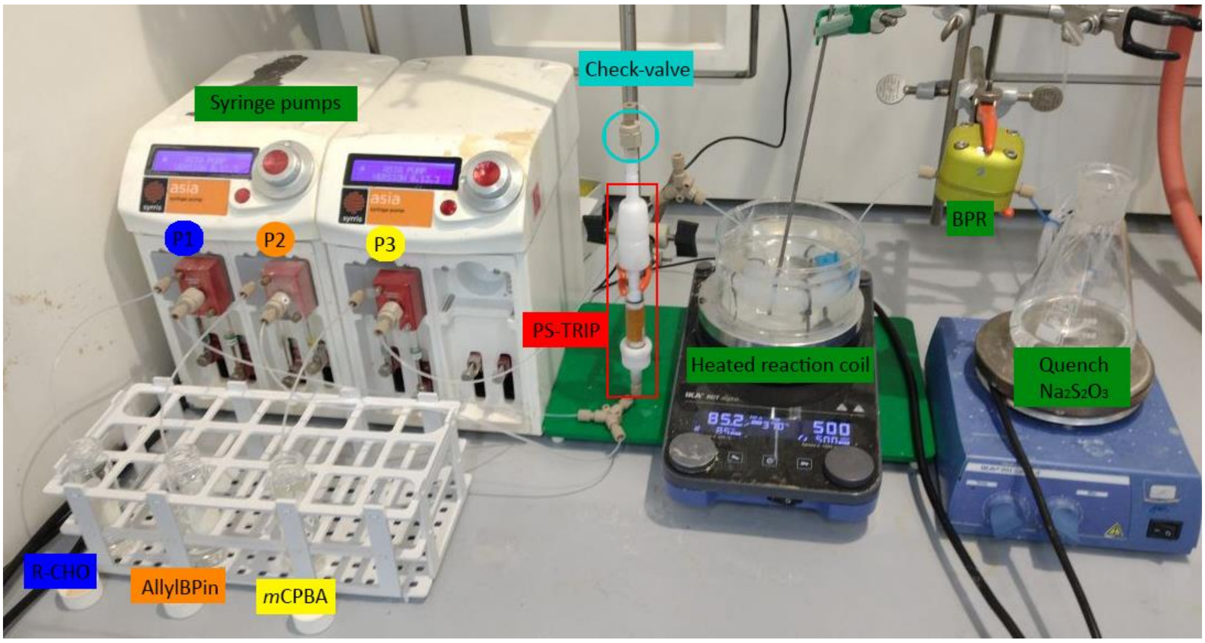

NMR and HPLC data of **4**, **5** and **7**

(*R*)-1-(thiophen-2-yl)but-3-en-1-ol (4c)

Racemic:

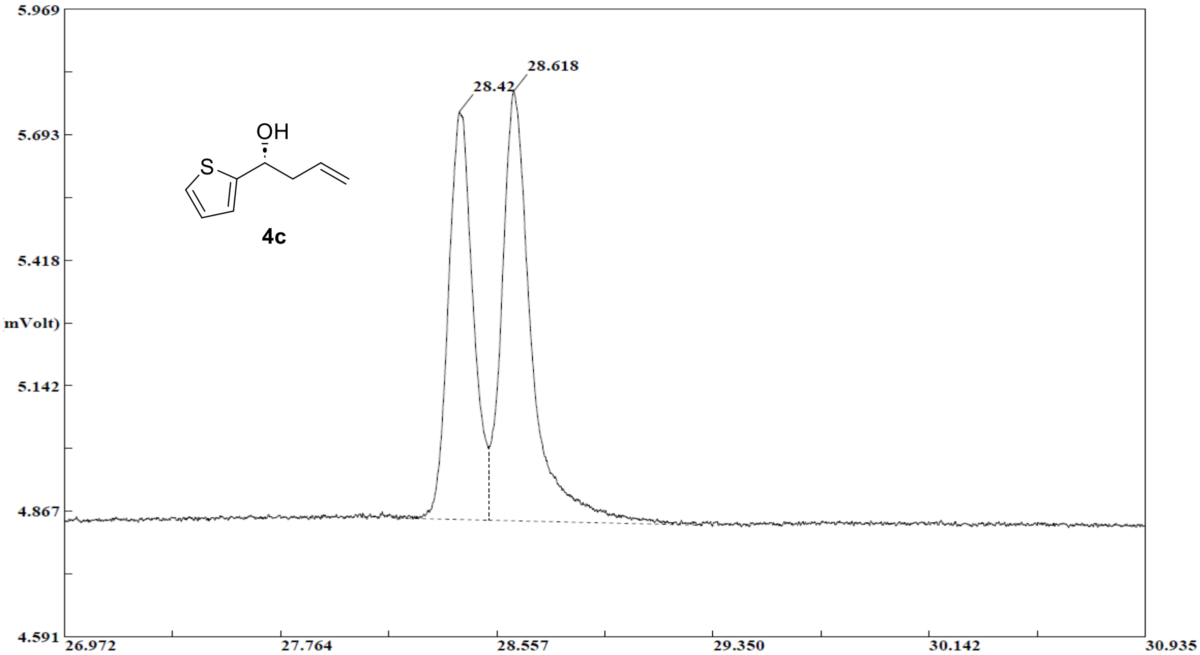

Peak Number #	Area %	Ret.Time	Area	BC
1	43.9273	28.42	56411	mi
2	56.0728	28.62	72008	mi
Totals	100.0000		128419	

Enantioselective: 66% ee

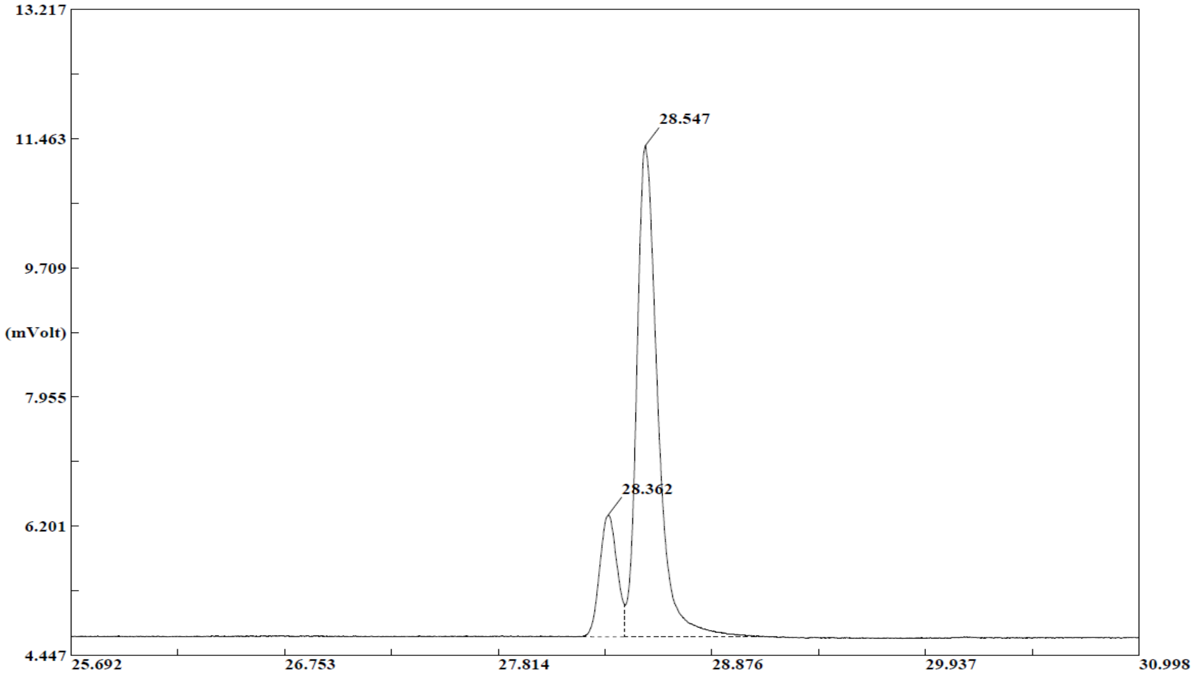

Peak Number #	Area %	Ret.Time	Area	BC
1	17.0216	28.36	99145	mi
2	82.9785	28.55	483321	mi
Totals	100.0000		582465	

(1*R*)-2-(oxiran-2-yl)-1-phenylethan-1-ol (5a).

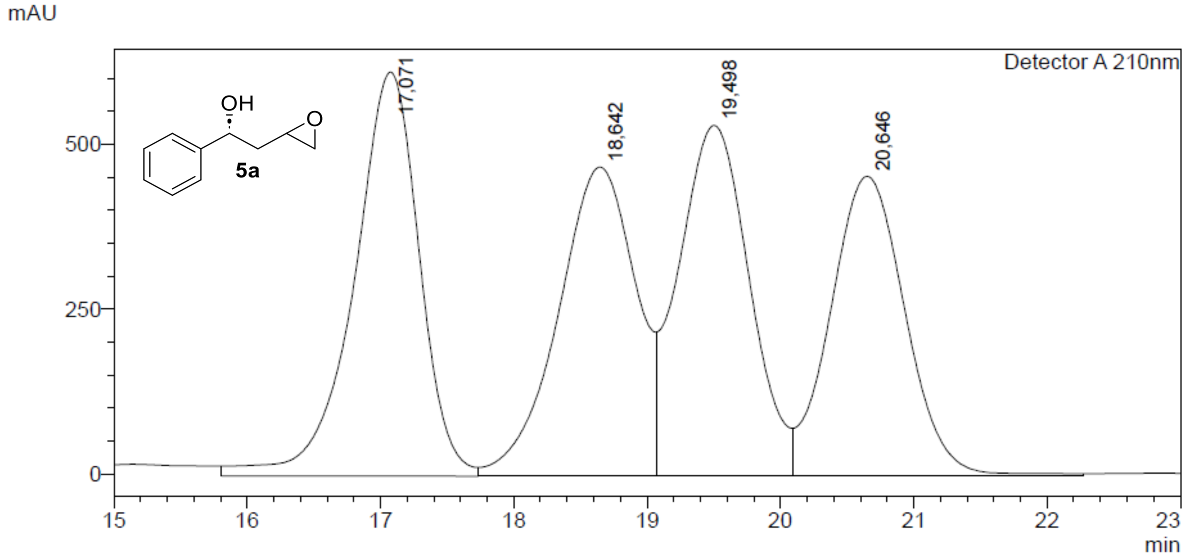

<Peak Table>

Detector A 210nm

Peak#	Ret. Time	Area	Height	Conc.	Name	Area%
1	17,071	20909152	613056	27,070		27,070
2	18,642	18889017	468868	24,455		24,455
3	19,498	19673074	532247	25,470		25,470
4	20,646	17768933	455147	23,005		23,005
Total		77240177	2069318			100,000

Enantioselective:

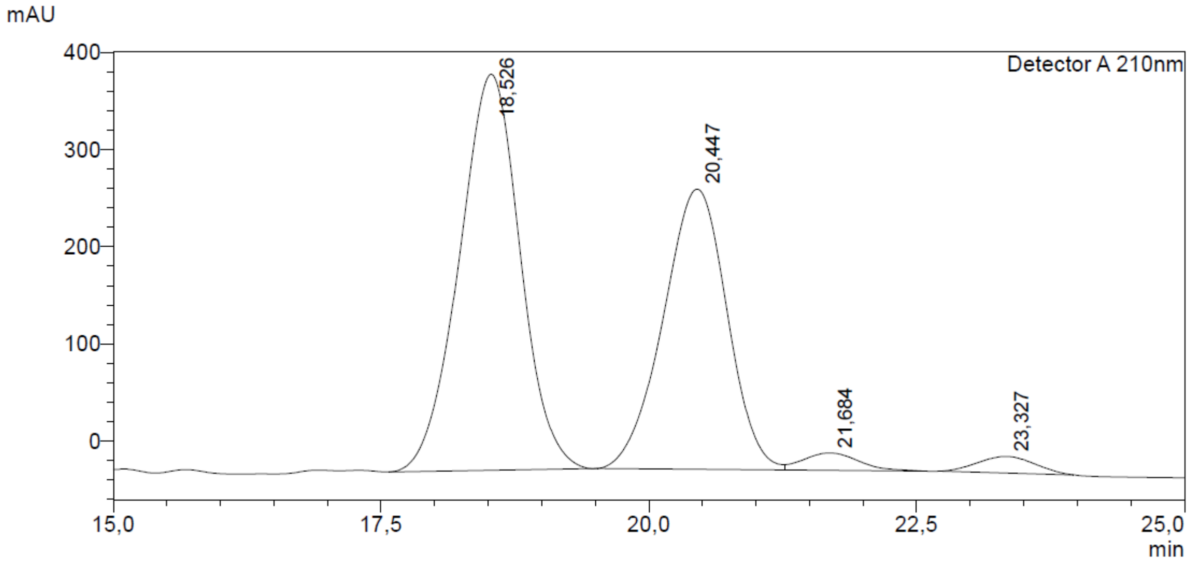

<Peak Table>

Detector A 210nm

Peak#	Ret. Time	Area	Height	Conc.	Name	Area%
1	18,526	16116466	407536	54,895		54,895
2	20,447	11956673	288350	40,726		40,726
3	21,684	652150	17958	2,221		2,221
4	23,327	633561	16966	2,158		2,158
Total		29358849	730810			100,000

Diasteromer 1: 92% ee

<Peak Table>

Detector A 210nm

Peak#	Ret. Time	Area	Height	Conc.	Name	Area%
1	18,526	16116466	407536	96,111		96,111
2	21,684	652150	17958	3,889		3,889
Total		16768615	425494			100,000

Diasteromer 2: 90% ee

<Peak Table>

Detector A 210nm

Peak#	Ret. Time	Area	Height	Conc.	Name	Area%
1	20,447	11652525	285265	94,992		94,992
2	23,327	614267	16704	5,008		5,008
Total		12266792	301969			100,000

(1*R*)-1-(4-fluorophenyl)-2-(oxiran-2-yl)ethan-1-ol (5b).

Racemic:

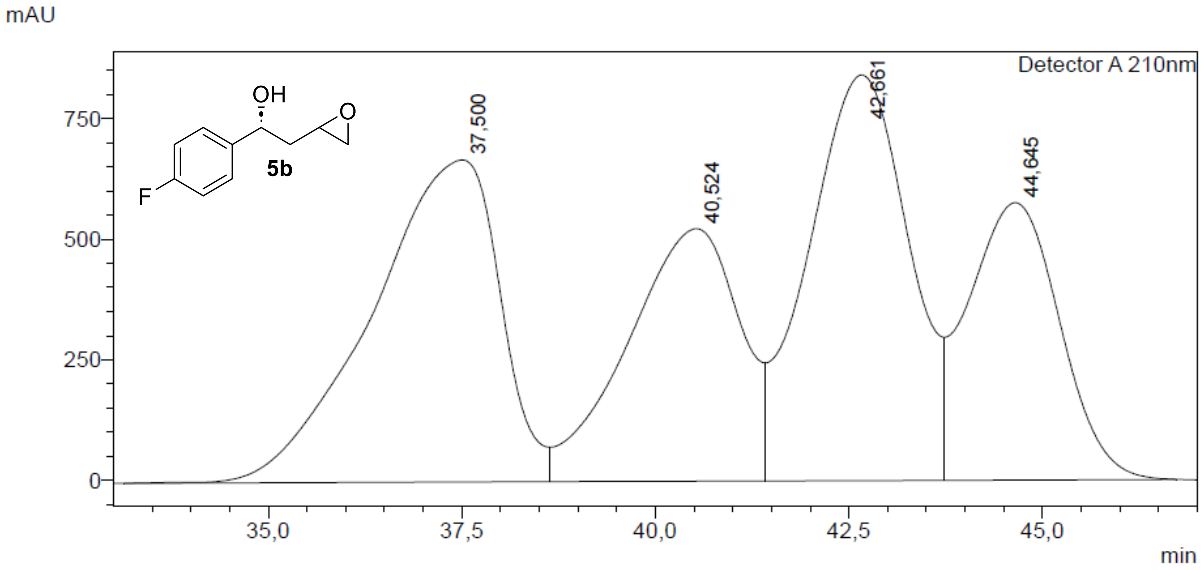

<Peak Table>

Detector A 210nm

Peak#	Ret. Time	Area	Height	Conc.	Name	Area%
1	37,500	76580489	667493	30,306		30,306
2	40,524	52683957	523248	20,849		20,849
3	42,661	76001884	840677	30,077		30,077
4	44,645	47423304	574651	18,767		18,767
Total		252689633	2606069			100,000

Enantioselective:

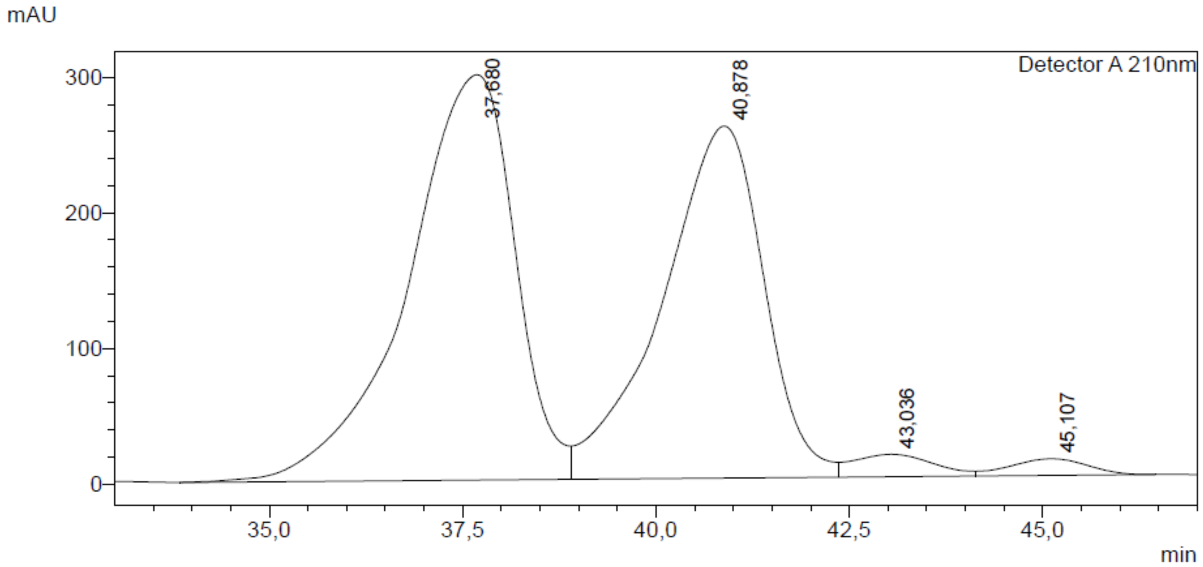

<Peak Table>

Detector A 210nm

Peak#	Ret. Time	Area	Height	Conc.	Name	Area%
1	37,680	28822619	298758	52,716		52,716
2	40,878	23800277	259469	43,530		43,530
3	43,036	1196435	16463	2,188		2,188
4	45,107	855808	12257	1,565		1,565
Total		54675140	586947			100,000

Diasteromer 1: 92% ee

<Peak Table>

Detector A 210nm

Peak#	Ret. Time	Area	Height	Conc.	Name	Area%
1	37,680	27684083	293003	95,767		95,767
2	43,036	1223634	15795	4,233		4,233
Total		28907717	308797			100,000

Diasteromer 2: 93% ee

<Peak Table>

Detector A 210nm

Peak#	Ret. Time	Area	Height	Conc.	Name	Area%
1	40,878	23434680	257960	96,477		96,477
2	45,107	855808	12257	3,523		3,523
Total		24290488	270218			100,000

(4*R*)-4-phenylbutane-1,2,4-triol (7a)

Racemic:

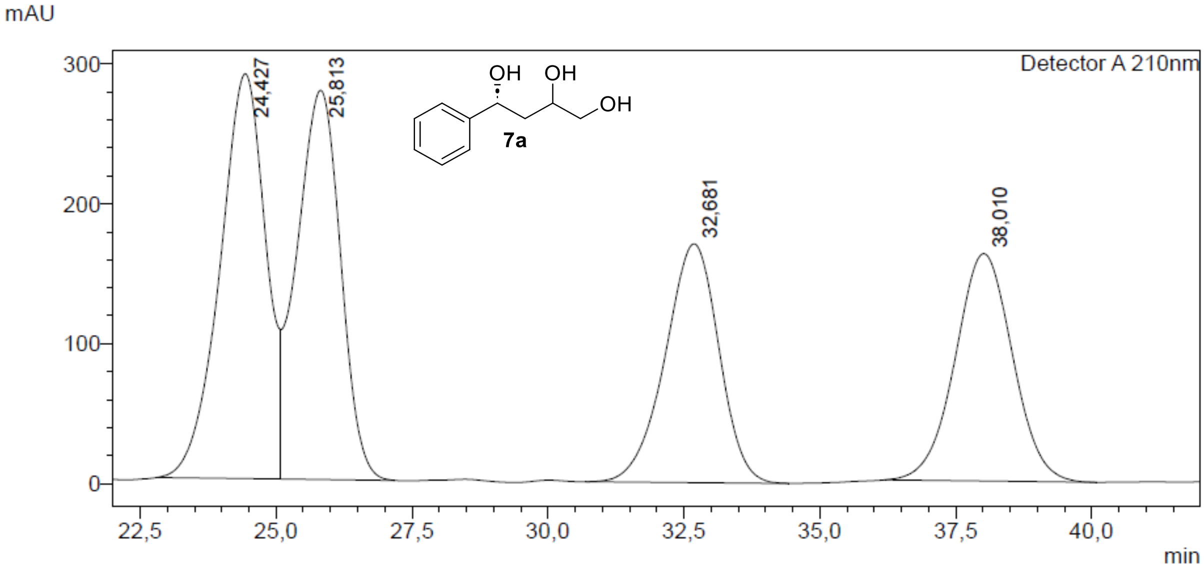

<Peak Table>

Detector A 210nm

Peak#	Ret. Time	Area	Height	Conc.	Name	Area%
1	24,427	16995806	289517	30,149		30,149
2	25,813	15643190	278338	27,750		27,750
3	32,681	11775056	170714	20,888		20,888
4	38,010	11957749	162604	21,212		21,212
Total		56371801	901173			100,000

Enantioselective:

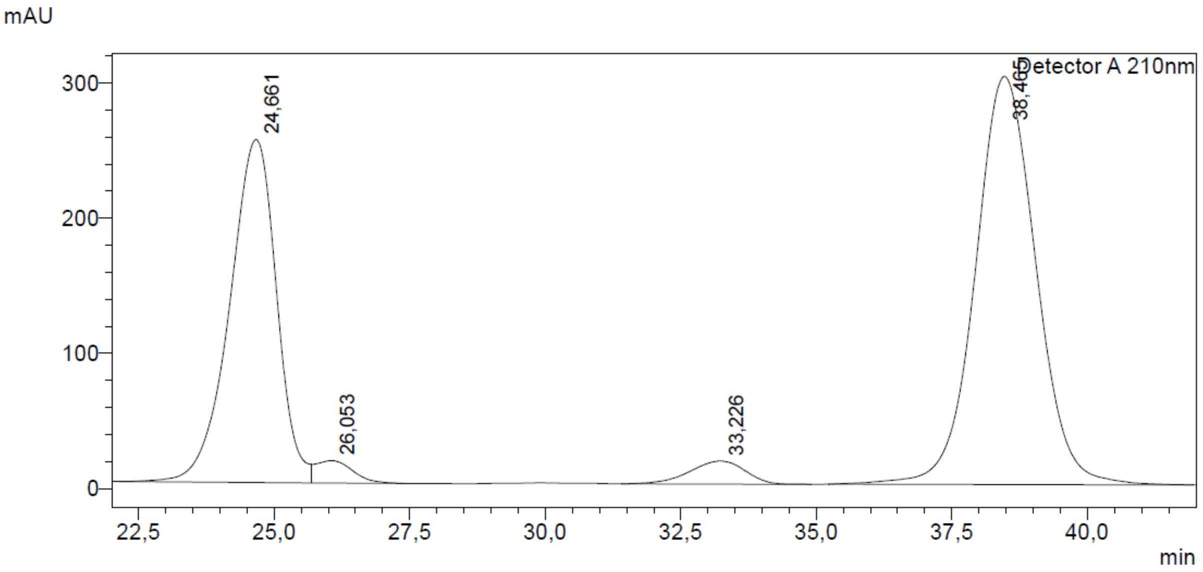

<Peak Table>

Detector A 210nm

Peak#	Ret. Time	Area	Height	Conc.	Name	Area%
1	24,661	15276772	253807	37,461		37,461
2	26,053	789511	16499	1,936		1,936
3	33,226	1220069	17062	2,992		2,992
4	38,465	23494651	301958	57,612		57,612
Total		40781004	589326			100,000

Diasteromer 1: 90% ee

<Peak Table>

Detector A 210nm

Peak#	Ret. Time	Area	Height	Conc.	Name	Area%
1	24,661	15276772	253807	95,086		95,086
2	26,053	789511	16499	4,914		4,914
Total		16066283	270306			100,000

Diasteromer 2: 90% ee

<Peak Table>

Detector A 210nm

Peak#	Ret. Time	Area	Height	Conc.	Name	Area%
1	33,226	1220069	17062	4,937		4,937
2	38,465	23494651	301958	95,063		95,063
Total		24714721	319020			100,000

NMR spectra of 4, 5 and 7

(*R*)-1-(thiophen-2-yl)but-3-en-1-ol (4c)

($CDCl_3$, ^1H 300 MHz)

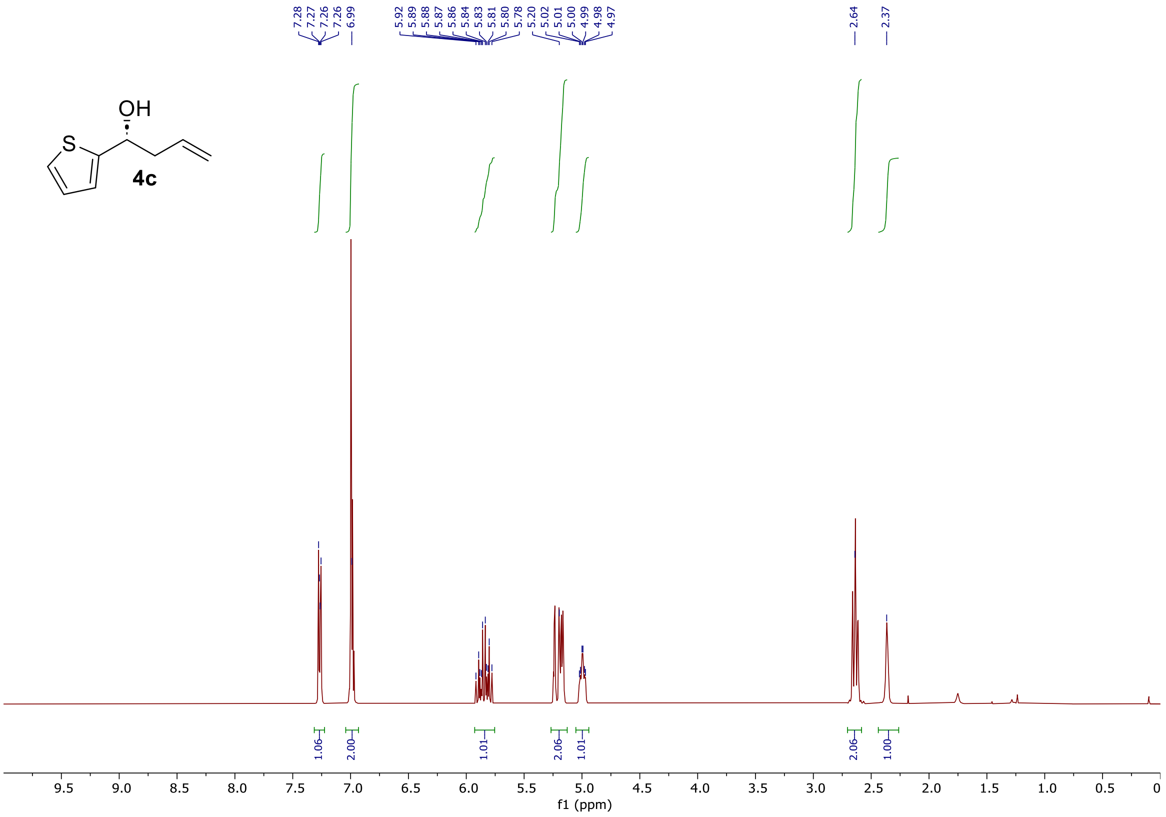

($CDCl_3$, ^{13}C {^1H} 75 MHz)

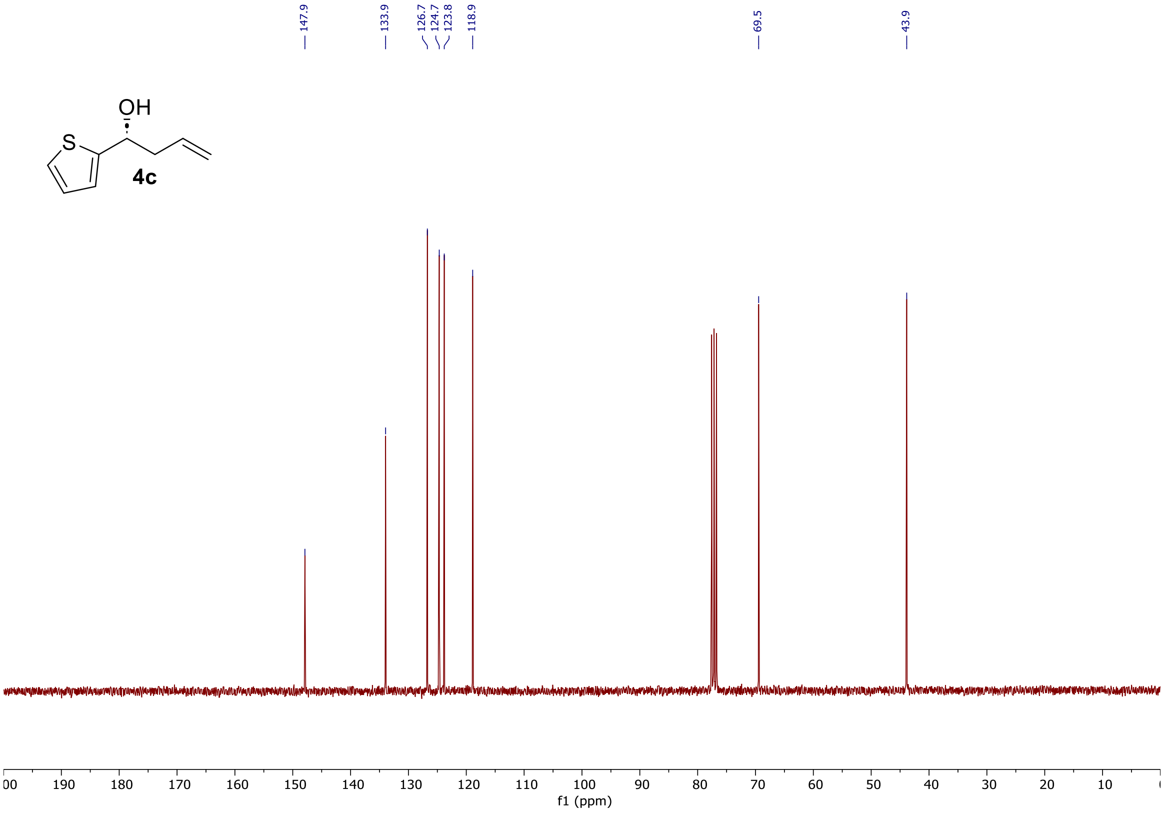

(1*R*)-2-(oxiran-2-yl)-1-phenylethan-1-ol (5a)

($CDCl_3$, ^{1}H 300 MHz)

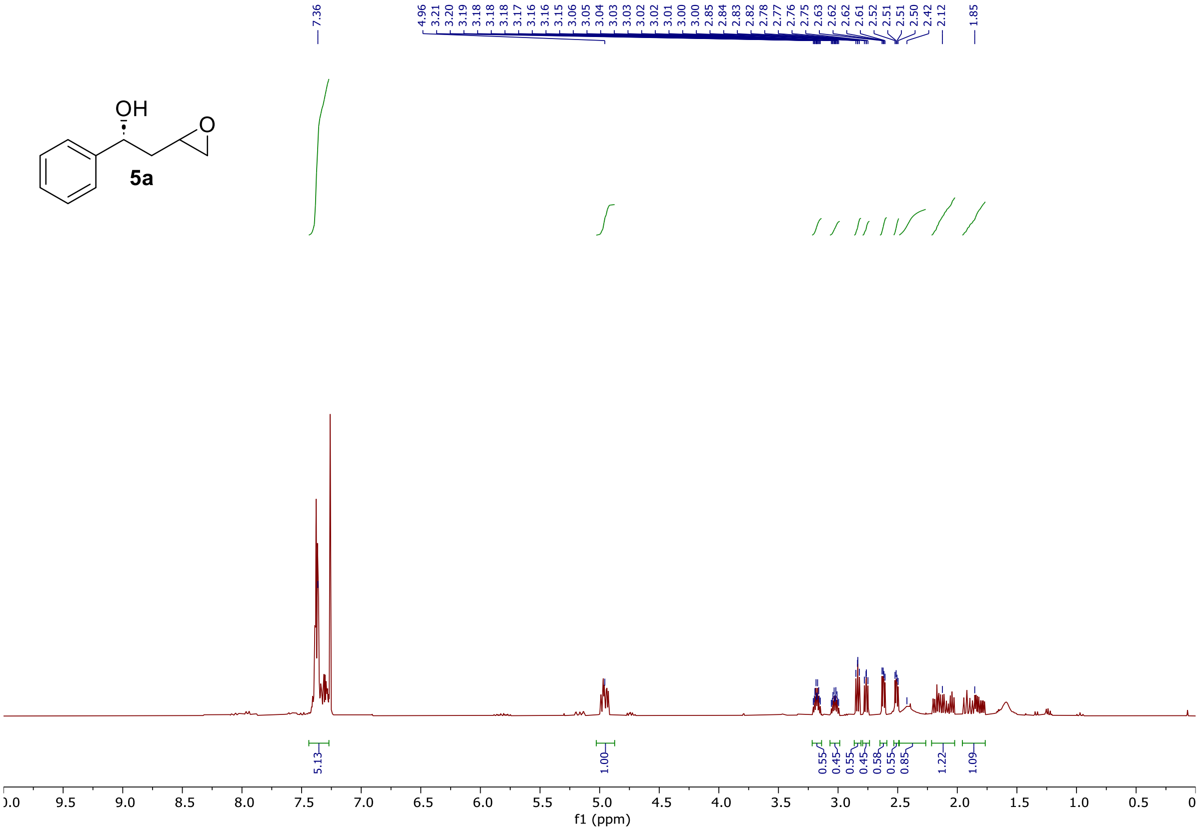

($CDCl_3$, ^{13}C {^{1}H} 75 MHz)

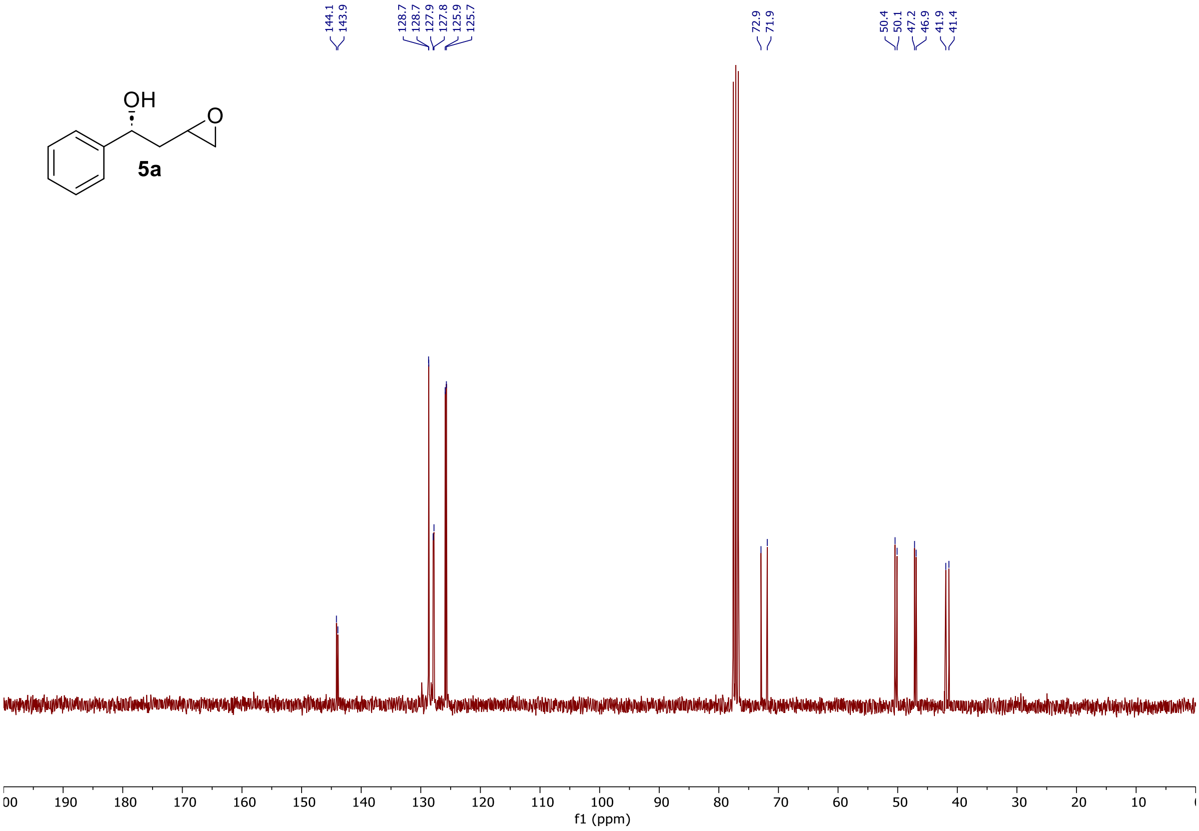

(1*R*)-1-(4-fluorophenyl)-2-(oxiran-2-yl)ethan-1-ol (5b)

(CDCl$_3$, ^{1}H 300 MHz, ^{13}C {})

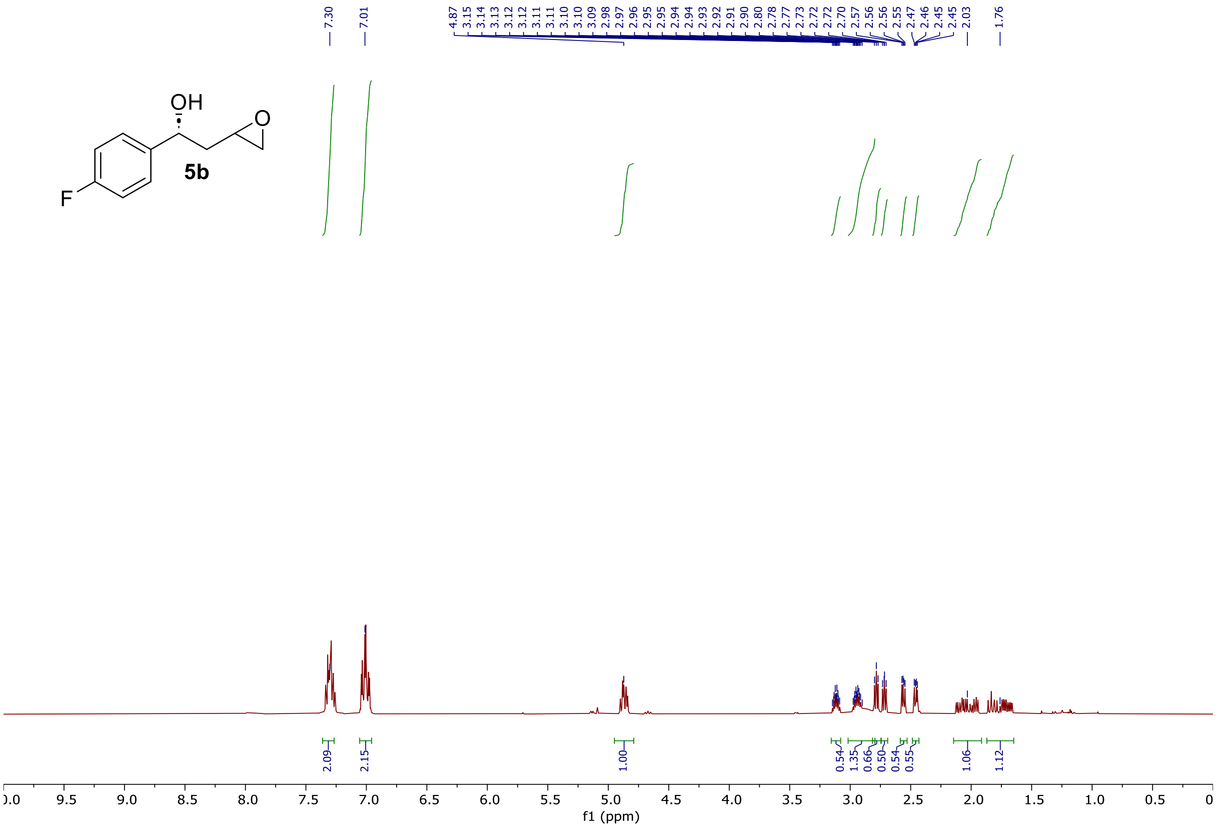

(CDCl$_3$, ^{13}C {^{1}H} 75 MHz)

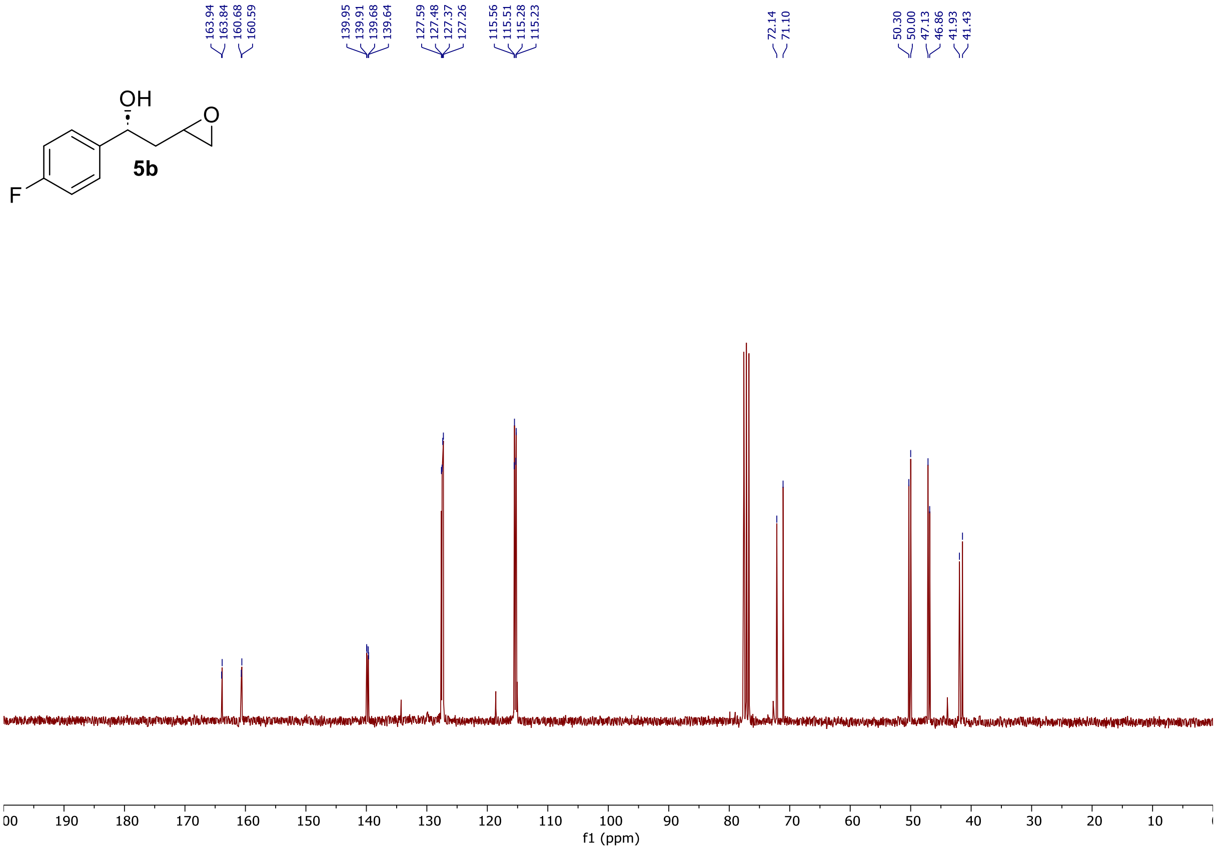

(CDCl$_3$, ^{19}F 282 MHz)

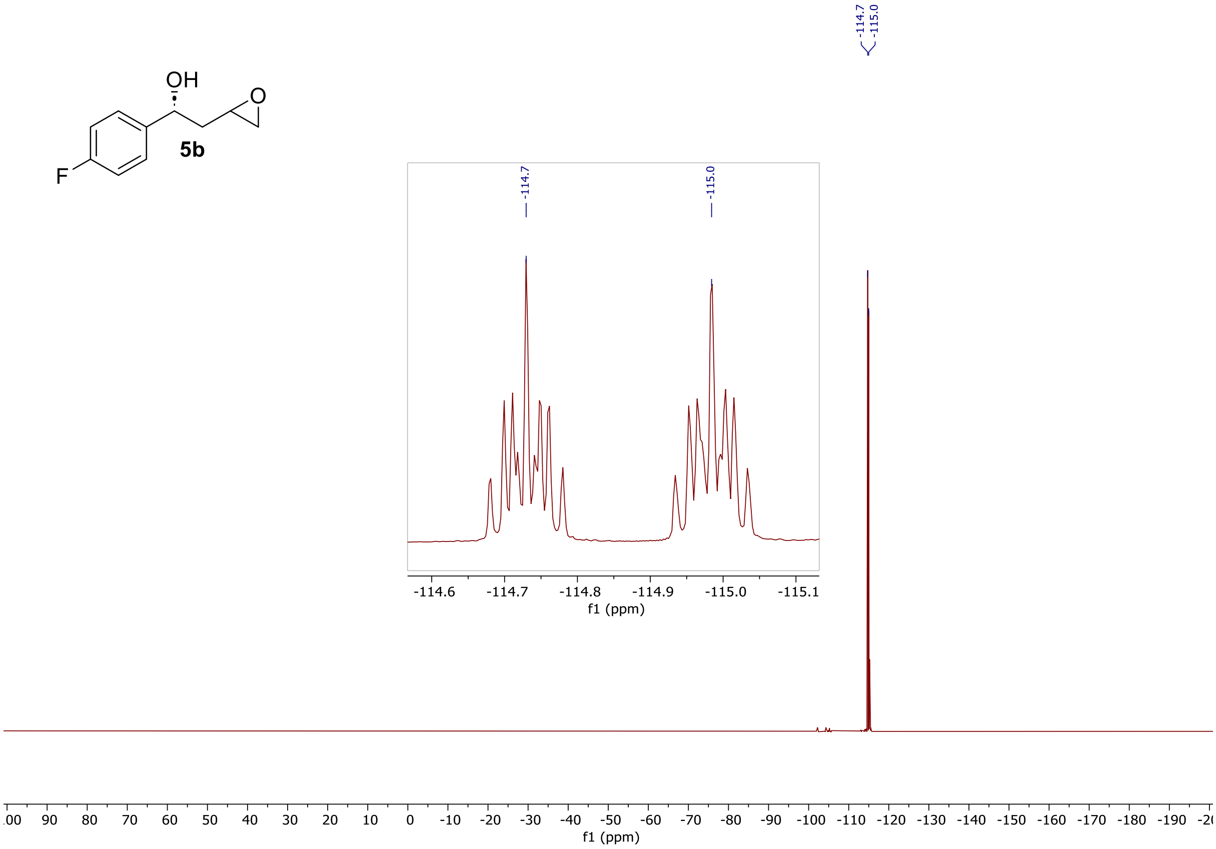

(4*R*)-4-phenylbutane-1,2,4-triol (7a)

($CDCl_3$, ^1H 300 MHz)

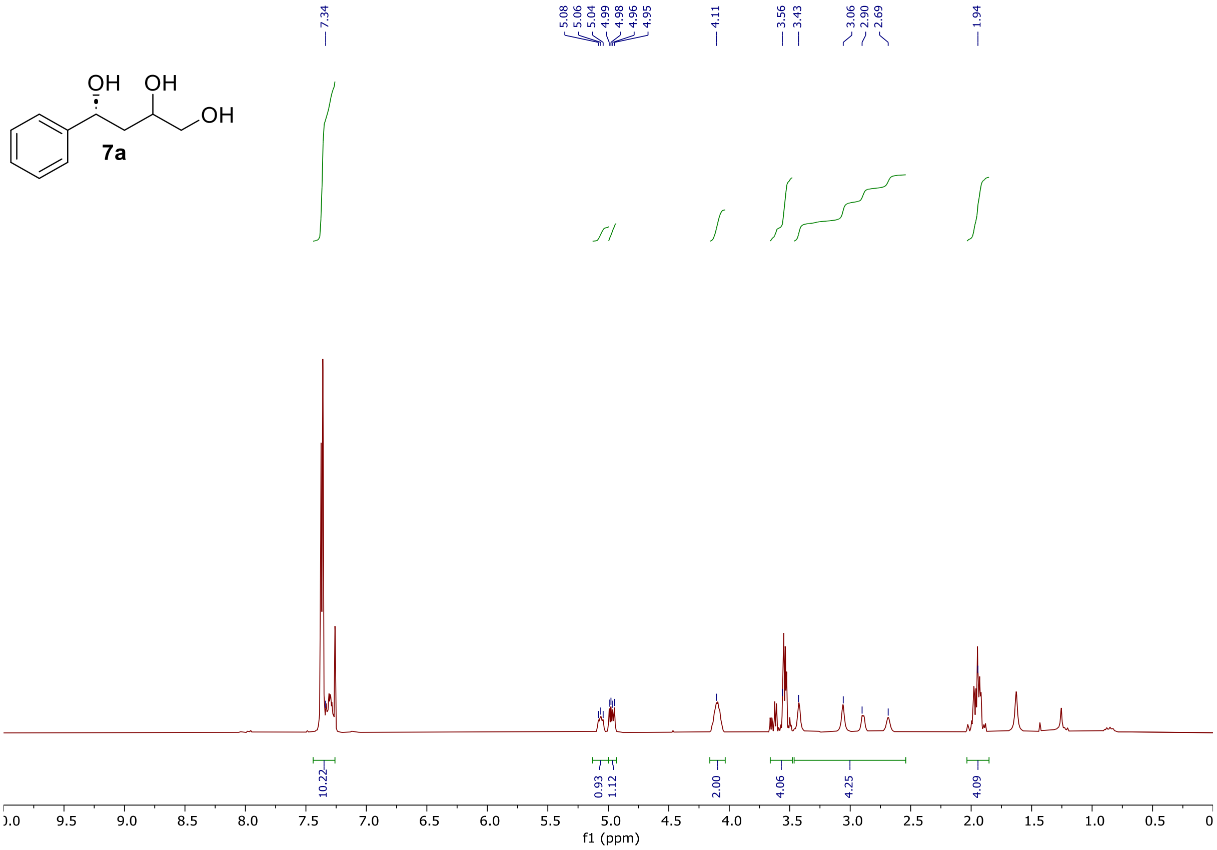

($CDCl_3$, ^{13}C {^1H} 75 MHz)

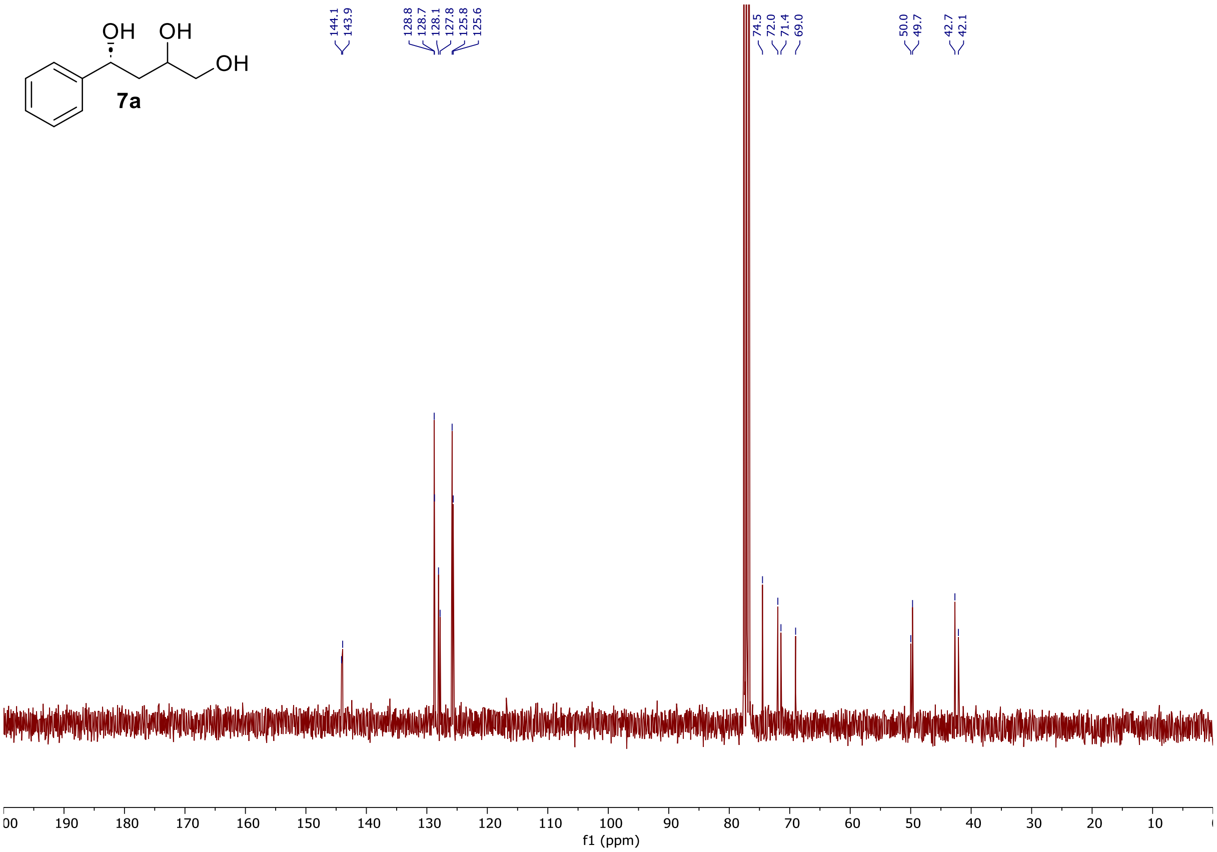

Supplement: Supplementary file 1 — jo3c02040_si_001.pdf [file jo3c02040_si_001.pdf]
